# Hemispheric specialization in selective attention and short-term memory: a fine-coarse model of left- and right-ear disadvantages

**DOI:** 10.3389/fpsyg.2013.00976

**Published:** 2013-12-24

**Authors:** John E. Marsh, Lea K. Pilgrim, Patrik Sörqvist

**Affiliations:** ^1^School of Psychology, University of Central LancashirePreston, Lancashire, UK; ^2^Department of Building, Energy, and Environmental Engineering, University of GävleGävle, Sweden; ^3^Linnaeus Centre HEAD, Swedish Institute for Disability Research, Linköping UniversityLinköping, Sweden

**Keywords:** ear-advantage, hemispheric asymmetry, distraction, short-term memory, left-ear disadvantage, right-ear disadvantage

## Abstract

Serial short-term memory is impaired by irrelevant sound, particularly when the sound changes acoustically. This acoustic effect is larger when the sound is presented to the left compared to the right ear (a left-ear disadvantage). Serial memory appears relatively insensitive to distraction from the semantic properties of a background sound. In contrast, short-term free recall of semantic-category exemplars is impaired by the semantic properties of background speech and is relatively insensitive to the sound's acoustic properties. This semantic effect is larger when the sound is presented to the right compared to the left ear (a right-ear disadvantage). In this paper, we outline a speculative neurocognitive fine-coarse model of these hemispheric differences in relation to short-term memory and selective attention, and explicate empirical directions in which this model can be critically evaluated.

One way in which our understanding of hemispheric specialization has been advanced is through the study of auditory processing (Cherry, 1953; Broadbent, 1958; Hugdahl, 2003; Hugdahl et al., 2009). Specifically, the combination of weaker ipsilateral pathways and stronger contralateral pathways within the auditory system results in the contralateral processing of sound. Input to the left ear, for example, has privileged access to the right hemisphere (RH), whereas input to the right ear has privileged access to the left hemisphere (LH). Sound, such as speech, is therefore predominately processed by the opposite hemisphere to its presentation source. These hemispheric differences result in the *right-ear advantage* in identifying or shadowing linguistic target-stimuli that are presented to the right-ear/LH (Kimura, 1961, 1967; Broadbent and Gregory, 1964; Studdert-Kennedy and Shankweiler, 1970) and the *left-ear advantage* for the processing of non-linguistic sound presented to the left-ear/RH (Tervaniemi and Hugdahl, 2003; Poeppel et al., 2004), especially with binaural sound presentation, although ear-advantages with monaural presentation have also been shown (Bradshaw et al., 1981). In this article, we review existing work on how this hemispheric asymmetry influences selective attention and short-term memory in the context of cross-modal auditory distraction.

## CROSS-MODAL DISTRACTION

### THE IRRELEVANT SOUND EFFECT (AND RIGHT HEMISPHERE PROCESSING)

The irrelevant sound effect refers to the observation that short-term memory for the correct serial order of a set of sequentially presented visual items (visual-verbal serial recall) is disrupted by the mere presence of background sound. Despite explicit instruction to ignore the sound, error rates can increase by up to 50% (Ellermeier and Zimmer, 1997). Two pre-requisites for irrelevant sound to produce substantial disruption are, first, that the focal task involves serial rehearsal of the to-be-recalled (TBR) items (Beaman and Jones, 1997), and second, that the irrelevant sound demonstrates appreciable acoustic variation from one sound element to the next (Jones and Macken, 1993). For example, if participants are required to maintain the serial order of TBR items, auditory changing-state sequences (e.g., “a b a b a b a”) are typically more disruptive than steady-state sound sequences (e.g., “a a a a a a a”), the *changing-state effect*. However, if participants are required to identify which member of a well-known set (e.g., Weekdays) that is not presented – the *missing-item task *– the changing-state effect does not emerge (Beaman and Jones, 1997). The theoretical reason for this is that the missing-item task does not require seriation of the TBR items, and so there is no conflict between the order information in the changing-state sequence and the processes that are required to fulfill the task. The combination of these two pre-requisites suggests that the changing-state effect is a function of the similarity between two sets of order processes: The deliberate processing of the order of the TBR items and the involuntary processing of the order between successive and perceptually discrete sound events (for a review, see Jones et al., 2010).

It does not matter whether the changing-state sequence consists of speech utterances or pure tones (e.g., Jones and Macken, 1993), the magnitude of disruption is rather a function of the sound’s acoustic variation (Jones et al., 2000), which suggests that the sound’s phonological and semantic content plays little if any role, although this is still the subject of debate (e.g., Bell et al., 2011). Item identity typically plays a more subservient role than acoustic variation (Jones et al., 2010), but serial recall of visual digits is more greatly impaired by irrelevant digits than irrelevant consonants if the order of the irrelevant digits is different (i.e., incongruent) to the order of the TBR digits, but not when it is similar (congruent; Hughes and Jones, 2005; Bell et al., 2011).

The changing-state effect has been used as an analytic tool to study hemispheric biases for passive processing of irrelevant sound. For example, Hadlington et al. (2004) found that speech utterances (Experiments 1a and 2a) and sine wave tones (Experiments 1b and 2b) impair serial memory to a greater extent when presented to the left-ear-only, relative to right-ear-only presentation and presentation to both ears. This *left-ear disadvantage* was later replicated in the context of a mental arithmetic task (cf. Banbury and Berry, 1998), but was not found in the context of a missing-item task (Hadlington et al., 2006). Moreover, the left-ear disadvantage was greater in magnitude when the irrelevant sequence conveyed acoustic variation, such as pitch changes, and varied inter-stimulus intervals, but it was absent when the sound stream contained little acoustic variation (such as a repetition of a single utterance). Collectively, serial short-term memory is more impaired from sound with a left-ear source, and it does not matter if that sound has lexico-semantic content or not.

These findings cohere nicely with the notion that the RH plays a prominent role in the obligatory processing of the acoustic features rather than the item identity/content within the irrelevant sound streams (Zatorre et al., 1994; Grimshaw et al., 2003; Poeppel et al., 2004): The RH specialization for processing serial information turns into a *disadvantage* when sound is to-be-ignored and the focal task also requires seriation.

### ITEM BASED DISTRACTION (AND LEFT HEMISPHERE PROCESSING)

As discussed above, acoustic variation appears to interfere selectively with serial memory in the RH, due to a conflict between deliberate order processes and an automatic analysis of acoustic change in the unattended sound. Both behavioral and neuroimaging studies propose that order and item information are supported by separate cognitive representations (for a review, see Marshuetz, 2005), which suggests that background sound could selectively impair item memory, just as it selectively impairs serial memory in the RH. This is the question we turn to next.

The LH appears to dominate language/semantic processing. For example, little lexical-semantic analysis of deliberately ignored speech is thought to take place in the RH (Beaman et al., 2007) and the LH responds to lexical-semantic information of auditory word stimuli (Zahn et al., 2000). Moreover, memory for verbal material is LH localized (e.g., Smith et al., 1996; Baddeley, 2003) and the right-ear advantage in dichotic listening (e.g., Hugdahl et al., 2009) supports privileged linguistic processing in the LH. Taken together, in the context of tasks that require semantic processing, which predominantly depend on the LH, background speech might actually be more disruptive when presented to the right ear. This hypothesis has recently received some support (Sörqvist et al., 2010).

Sörqvist et al. (2010) used a paradigm wherein TBR visual lists comprise exemplars that are members of the same semantic category (e.g., Fruit). To-be-ignored spoken words that are taken from the same semantic category as the TBR items (e.g., other Fruit) produce greater disruption to free recall than to-be-ignored words from a different semantic category (e.g., Tools): the *between-sequence semantic similarity effect* (Marsh et al., 2008). This effect is indexed as fewer correct recalls of visual-targets and greater false recall (e.g., of words that were spoken in the background). The between-sequence semantic similarity effect is found when speech is presented to the right-ear/LH but not when it is presented to the left-ear/RH (Sörqvist et al., 2010). Importantly, this *right-ear disadvantage* is only found when the task is to recall the items in *free *order (Experiments 1 and 3), not when they must be recalled in order of presentation**(Experiment 2).

Thus, task requirement appears to determine when a left-ear or a right-ear disadvantage is found. Verbal item memory, localized to the LH, is more impaired when task-irrelevant linguistic information is presented to the right-ear/LH, whereas serial order memory, predominantly localized to the RH, is more impaired by acoustically varying sound presented to the left-ear/RH. Interestingly, the ear-*disadvantages* have been shown in the context of monaural sound presentation, whereas the ear-*advantages* are typically found with binaural presentation. A right-ear advantage is found with monaural presentation, however, when several sound streams are presented simultaneously to the same ear, and there is a need to resolve stimulus competition (Bradshaw et al., 1981). Taken together with the cross-modal effects, the ear asymmetries in monaural presentation may arise because of the competition between processing streams.

#### False recall

In the context of free recall, spoken words that are related (e.g., Tools) to the TBR items (e.g., Tools) typically produce more false recall (of items that belong to the target category, but were not part of the target list) than unrelated spoken words (e.g., Occupations; Marsh et al., 2008). This effect is greater for right-ear/LH presentation (Sörqvist et al., 2010). Initially, these results appear consistent with the model offered by Beaman et al. (2007) wherein it is assumed that right-ear input increases the capacity of speech to interfere with the semantic processes in the LH, whereas this capacity is attenuated for left-ear input. However, Sörqvist et al. (2010) found that *unrelated* speech presented to the left-ear/RH resulted in *more* false recall than unrelated speech presented to the right-ear/LH (i.e., a left-ear disadvantage for the effect of unrelated speech on false recall). Moreover, despite generation of fewer intrusions with unrelated speech presented to the right-ear/LH, those that emerged were generally greater in output-dominance (e.g., DOG is a more dominant member than LIZARD of the category “four-legged animal”). The finding that *unrelated* speech presented to the left-ear/RH has systematic effects on false recalls suggests some lexical-semantic processing of irrelevant speech within the RH.

## UNDERSTANDING THE PATTERN OF INTRUSIONS ACROSS THE HEMISPHERES

Hemispheric asymmetries in (attended) semantic processing are well documented. For example, Beeman and colleagues (Beeman et al., 1994; Beeman and Chiarello, 1998; Bowden and Beeman, 1998; Beeman and Bowden, 2000; Bowden and Jung-Beeman, 2003) suggest that the LH is particularly attuned to fine processing, activating a restricted semantic network comprising of a small number of closely related concepts. In contrast, the RH specializes in coarse processing (Tervaniemi and Hugdahl, 2003) activating a widespread, diffuse array of associates.

This fine-coarse processing mechanism is supported by evidence from semantic priming. Greater summation priming from three weakly-related, centrally-presented, prime words (e.g., *foot*-*cry*-*glass*) is found when the target word (e.g., *cut*) is presented to the left visual-field/RH as opposed to the right visual-field/LH (Beeman et al., 1994). In contrast, directly related primes (e.g., *scissors*) show greater priming than summation primes when targets are presented in the right visual-field/LH. The idea is that the RH weakly activates large semantic fields, which overlap, and therefore, although each semantic field is only weakly activated, this overlap allows the weakly related concepts to activate more strongly, reaching threshold. In contrast, the LH strongly activates narrow semantic fields, activating only dominant meanings, or meanings that are most relevant to the immediate context.

The novel approach that we take here is to attempt to explain how Beeman et al.’s (1994) fine-coarse model can account for the findings that (a) an *unrelated* stream of words presented to the right-ear/LH suppresses false recall, and that (b) the intrusions produced when *unrelated *words are presented to the right-ear/LH are greater in output-dominance (Sörqvist et al., 2010). One possible explanation for these findings, that concerns false recalls, can be found in relation to how hemispheric differences in semantic activation influences selection of candidates for recall.

Semantic activation across the hemispheres is defined in terms of: speed, strength, and breadth. In the LH, semantic activation quickly focuses in on a narrow semantic field of strongly activated items relevant to the current task. In contrast, the pattern in the RH is more diffuse and weak, activating a broad semantic field of both more and less relevant related items. These different patterns of activation across the hemispheres (LH: quick, small, strong versus RH: slow, broad, weak), are likely to result in a different level of false recall. We expand on this point below.

### LEFT HEMISPHERE PRESENTATION

#### Unattended related items

**FIGURE 1:**
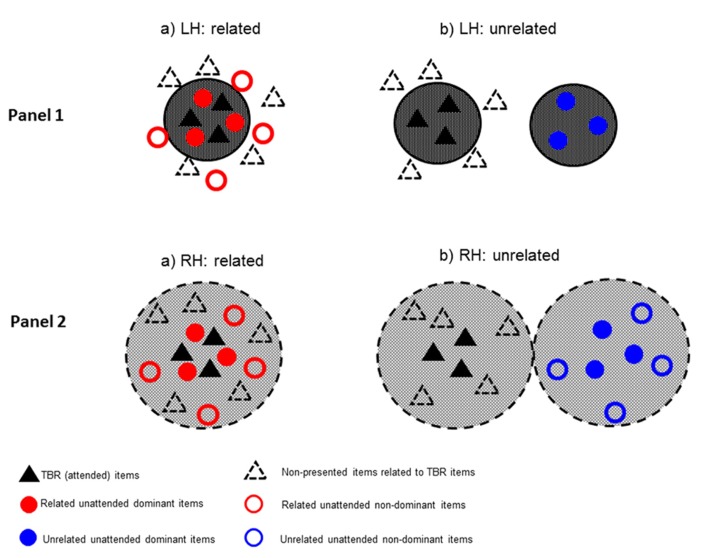
**The figure shows the fine-coarse model of semantic activation when TBR and unattended items are presented to the right-ear/LH (Panel 1) or left-ear/RH (Panel 2), and when the unattended items are either semantically related (a) or sematically unrelated (b)**.

Strong activation quickly narrows down to focus on the cohort of relevant items (i.e., the TBR items). Some of the unattended related items would also fall within this narrow semantic field (**Figure 1**, Panel 1a). Connectivity between all these related items would likely boost levels of activation within the entire cohort. Furthermore, unattended items that are activated are likely to be the ones that are most closely related to the TBR items. In contrast, more distantly related items are likely to be outside this core semantic network and, thus, would not benefit from the strong activation and interconnectivity between items. This is likely to result in high levels of intrusion from closely related (dominant) items but with little interference from (and hence false recall of) distantly related (non-dominant) items.

#### Unattended unrelated items

The TBR and unattended unrelated items activate two separate semantic networks (**Figure 1**, Panel 1b). However, unlike the related condition, there would be no connectivity between TBR and unattended items and thus little interference. As with the unattended related items, false recall would likely be confined to dominant items closley related to the TBR items.

### RIGHT HEMISPHERE PRESENTATION

#### Unattended related items

The RH weakly activates a broad, diffuse semantic network encompassing both TBR and unattended related items. Although intrusions are less likely than in the LH (due to weaker activation), the wide network of interconnected related items suggests that some unattended items are likely to reach a threshold where false recall is possible. Due to the broad semantic network activated, these intrusions would be equally likely from either closely related (dominant) or weakly related (non-dominant) items (**Figure 1**, Panel 2a). In addition, the diffuse activation makes it possible that related, but non-presented items, are also activated.^1^ However, because of the strong competition from the mutally activating TBR and unattended items, non-presented items are unlikely to reach threshold for intrusion.

#### Unattended unrelated items

The TBR and unattended unrelated items activate two separate semantic networks (see **Figure 1** Panel 2b). Thus, unlike the unattended *related* items, the unattended *unrelated* items do not benefit from mutual activation via the TBR items. This would result in them having less chance of reaching threshold, as they receive no boost from interconnectivity with the TBR items. However, intrusions in the unattended unrelated condition would still be more likely in the RH than the LH, because the broad semantic network allows a greater connectivity between cohort members than in the LH, resulting in increased levels of activation for some items within the cohort. As in the unattended related condition, intrusions would be equally likely from either closely related (dominant) or weakly related (non-dominant) items. Finally, intrusions from non-presented items that are related to the TBR items may be slightly higher than in the unattended related condition, because they benefit from connnectivity with the TBR items, but do not suffer from additional competition from the unattended items.

## EXTENSIONS AND PREDICTIONS OF THE MODEL

The fine-coarse model of hemispheric specialization supposes that the retrieval information from semantic memory (a process that underpins short-term memory for identity) will be vulnerable to disruption via meaningful speech presented to the right-ear/LH whereas the process of serial rehearsal (a process that underpins short-term memory for order) will be more impaired by acoustically variable sound presented to the left-ear/RH. Moreover, the model suggests that the ways in which background speech promotes false recall will depend on the semantic relation between targets and distracters and the dominance of the distracters. In general, the empirical findings presented here are consistent with the fine-coarse model.

The concept of hemispheric asymmetry in processing suggested by the fine-coarse model can be theoretically useful in informing the debate between interference-by-process (Jones and Tremblay, 2000; Marsh et al., 2009) and interference-by-content (Salamé and Baddeley, 1982, 1986; Neath, 2000) accounts of auditory distraction within short-term memory. The findings reviewed here are at odds with the interference-by-content approach whereby the irrelevant sound effect is viewed as a function of the similarity in identity between the TBR and irrelevant items. In contrast, the findings with by-ear presentation harmonize with the more dynamic interference-by-process account according to which the type of distraction that takes place (item or order based distraction) does not depend on the materials of the focal task but on the nature of the cognitive operations that are carried out to process that material. Here, we outline ways in which the fine-coarse model can be used to further explore this distinction between item and order based distraction.

### FREE RECALL

One way to test the predictions of the fine-coarse model, as outlined, is through manipulating the output-dominance of the unrelated speech within free recall. Low output-dominant items that are weakly representative of their category should result in more activation – and hence promote more false recalls – when presented to the left-ear/RH in comparison with presentation to the right-ear/LH. A smaller by-ear effect should be found for the presentation of unrelated speech that conveys high output-dominant category members.

### SERIAL RECALL

As noted, similarity in item identity between target and distracters can play a role in disruption of serial recall (Hughes and Jones, 2005). This can be further explored through manipulating the size of the TBR item set. For example, letters come from a wider set (26 in English) than digits (0–9) and thus the burden on item memory can be greater with letters. By-ear presentation could yield some clues as to whether some variants of the serial recall task simply tap into item-based effects. Specifically, the role of the RH (and therefore the left-ear disadvantage) should be much reduced (and possibly even turn into a right-ear disadvantage) when the serial recall task comprises a larger set (e.g., 8 of 26 items presented on any given trial). A further extension along these lines would be to investigate the role of individual differences. Individual differences in working memory capacity are unrelated to the magnitude of the changing-state effect (Sörqvist et al., 2013), but related to the ability to resist attention capture (Sörqvist, et al., 2010) and to semantic effects (Beaman, 2004). As there are also substantial individual differences in ear-advantages (Hugdahl, 2000), perhaps the role for item-based disruption in the context of serial memory can be further explored by analyzing the relation between working memory capacity (WMC) and ear-disadvantages. Indeed, Beaman et al. (2007) suppose that WMC is associated with the capability to modify the activity or output from lexico-semantic analysis in the left superior temporal gyrus (STG).

## Conflict of Interest Statement

The authors declare that the research was conducted in the absence of any commercial or financial relationships that could be construed as a potential conflict of interest.

## Footnotes

1 In contrast, in the LH it is unlikely that non-presented items would be activated due to the narrow semantic field activated (see in **Figure 1** Panel 1).
